# Relative Health Risk Reduction from an Advanced Multi-Modal Air Purification System: Evaluation in a Post-Surgical Healthcare Setting

**DOI:** 10.3390/ijerph21081089

**Published:** 2024-08-17

**Authors:** Dino Pisaniello, Monika Nitschke

**Affiliations:** School of Public Health, University of Adelaide, Adelaide, SA 5005, Australia; monika.nitschke@adelaide.edu.au

**Keywords:** air purification, ventilation, airborne particle, indoor air quality, healthcare, intervention, infection risk, microbiological hazard, airborne disease transmission

## Abstract

Advanced air treatment systems have the potential to reduce airborne infection risk, improve indoor air quality (IAQ) and reduce energy consumption, but few studies reported practical implementation and performance. PlasmaShield^®^, an advanced multi-modal HVAC-integrated system, was directly compared with a standard MERV-13 system in a post-surgical paediatric healthcare setting. The evaluation entailed monitoring of multi-size airborne particles, bioaerosols and key IAQ parameters. Measurements were taken for outside air, supply air and air in the occupied space for 3 days prior to, and after, the installation of the PlasmaShield system. Compared with the existing arrangement, very significant reductions in particle number concentrations were observed in the occupied space, especially with virus-like submicron particles. Significant reductions in airborne culturable bacteria and fungi were observed in the supply air, with more modest reductions in the occupied space. In the case of virus-like particles, there was an eight-fold improvement in equivalent clean air, suggesting a five-fold infection risk reduction for long-range exposure. The data suggest multiple benefits of airborne particle and bioaerosol reduction, with applications beyond healthcare. Long-term studies are recommended to confirm the combined IAQ, health and energy benefits.

## 1. Introduction

Around the world, experts from various disciplines are calling for a comprehensive reconsideration of indoor air quality (IAQ). High health-related costs from infectious and chronic respiratory disease and human performance loss associated with a variety of indoor air contaminants were already being discussed prior to the COVID-19 pandemic [1]. Now, the need for clean indoor air has culminated in calls for action regarding the indoor environment, akin to what once was achieved for safe drinking water many decades ago [2].

The significant contribution of building air conditioning to global greenhouse gas (GHG) emissions adds to the concern. Thus, there is now the triple challenge of improving IAQ, reducing airborne viral disease transmission and reducing the carbon footprint of heating, ventilation and air conditioning (HVAC) systems [3]. In their scoping review, Bolten and coworkers reported that healthcare is a sector globally responsible for 4.4% of annual GHG emissions, and 7–10% in high-income countries; HVAC systems in hospitals account for up to 40% of emissions [3]. Energy-efficient air purification systems, based on multiple modes of action to deal with various air contaminants including toxic particles and volatile organic compounds (VOC), are required to meet this triple challenge.

During the first year of the COVID-19 pandemic, the airborne pathway was gradually accepted, recognising that infectious particles in the range of <5 micrometres (μm) can stay in the air and impact over large distances [4,5,6]. This also applies to a range of other infectious particles that are transmitted via the airways including influenza, tuberculosis, measles and many more [7]. Recently, the increased risk of hospital-acquired COVID-19 infections has been shown, which is concerning for staff and vulnerable patients [8,9].

Historically the main functions of building HVAC systems were to provide occupant thermal comfort and to introduce fresh air from the outside, which assists in alleviating odours and the build up of environmental pollutants. In the context of infectious aerosols and COVID-19, international ventilation guidelines were developed [10,11]. While there are no specific legislated standards in Australia, the Victorian Department of Health suggests that building systems should attain more than six air changes per hour (ACH) to achieve a meaningful reduction or dilution in the numbers of particles, including viruses such as SARS-CoV-2 [12]. For standard hospital rooms, a minimum of 5 ACH is required and for negative-pressure isolation rooms, 12 ACH [12].

An increase in ventilation can assist in reducing the spread of infectious disease, but it cannot eliminate the risk of airborne diseases, for example by close range ballistic exposure [13]. Increasing the amount of outside air leads to significantly higher energy usage and GHG emissions, along with increased implementation costs. These factors create obstacles to its practical application in many scenarios. Some healthcare spaces have even more stringent requirements such as high-efficiency particulate air (HEPA) filters either in-built into the HVAC systems or as a freestanding room air cleaner with HEPA filters. These HEPA filters have the capacity to filter out 99.97% of particles, down to the size of particles of 300 nm as defined in the Australian Standard AS 4260 [14]. The HEPA filter extends its usefulness to non-infectious particles likely to reach the indoor environment such as pollen and fungi and air pollution-related fine particulate matter, all of which have been reported to cause health effects [15].

However, there are limitations to HEPA filtration alone. The energy requirement for air to be pumped through the restrictive filters is extremely high, as is the requirement for exchanging the filters regularly as they pose a potential health hazard in relation to viable microorganisms. Direct evidence of virus-containing aerosols trapped on a portable air cleaner HEPA filter has been reported [16].

The Australian Commission on Safety and Quality in Health Care provides guidance in relation to optimising the mechanical ventilation and filtration systems for infection prevention and control in healthcare settings but has yet not extended its guidance to a system combining ventilation and filtration with an added-on disinfection system [17]. This is most likely due to the reality that emerging air disinfection techniques have not yet been trialled for their efficiency in real life settings. Recently, there have been advanced prototype investigations into air disinfection using UV-C techniques providing good results [18]. Advanced HVAC-integrated air purification systems have been reported in comparative studies, and these have seen a reduction in hospital stays [19,20]. Arikan and coworkers reported a reduction in surface microbial levels following air treatment in an intensive care unit [21]. There was a significant positive correlation between the number of colonies detected and the rate of hospital-acquired infections. However, the evidence base is incomplete [22], and further trials are under way [23].

PlasmaShield^®^ is a medical-grade multi-modal air purifier fitted to a new or existing HVAC system. Laboratory tests demonstrate effective particle removal and the mitigation of microbial contamination via multiple modes of action (electron beam irradiation, electric field electroporation and final filtration) [24,25]. More specifically, the electrons penetrate cell walls to inactivate viruses, the electric field ruptures cell membranes and the final filtration entails a medium-efficiency particle filter and a low-resistance catalyst filter incorporating metal oxides.

This study evaluates the PlasmaShield technology in the real-life situation of a post-surgical recovery suite within a paediatric hospital in Adelaide, South Australia. The aim of the study was to compare, in an intervention trial design, its performance with a pre-existing system, based on minimum efficiency reporting value (MERV) 13 filtration [26]. The evaluation entailed multi-size airborne particle monitoring, and cultural bacteria and fungi assessment, as well as monitoring of key IAQ parameters.

## 2. Materials and Methods

### 2.1. Study Location

The Women’s and Children’s Hospital was the location for the intervention study. Following uncomplicated surgery in adjacent operating theatres, mothers and newborn babies are transferred to beds in a recovery suite, where they are kept under observation and care by nursing staff. Infection prevention and control is an imperative. The suite is operational from about 7.30 a.m. to 4.30 p.m., although staff are generally present for slightly longer periods of time.

**Figure 1:**
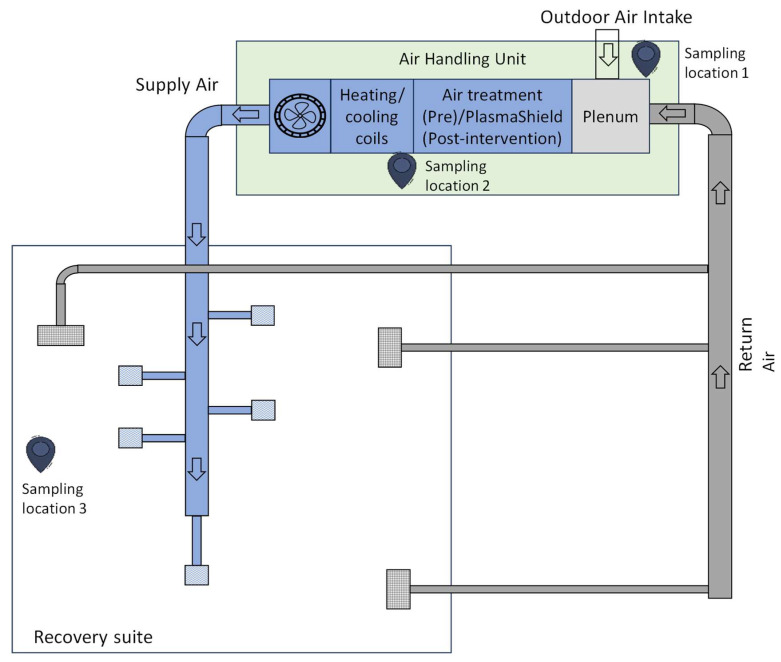
Ventilation layout of the recovery suite with air sampling locations 1–3: outdoor air, supply air and recovery suite room air.

Figure 1 is a simplified (not-to-scale) schematic diagram of the HVAC system layout.

It also indicates the three sampling points for (1) outdoor air intake, (2) supply air and (3) room air. Sampling location 1 is immediately adjacent to the air handling unit (AHU). Sampling location 2 is within the AHU after the PlasmaShield or MERV-13 filter.

Sampling location 3 is in the recovery suite, approximately 17 m from sampling locations 1 and 2, and at about a 1.5 m height. The outside air is drawn from the plant room, which is adjacent to the recovery suite. Additional outside air intake feeds into the plant room. This intake is about 20 m from the plant room, and at the perimeter of the building.

### 2.2. Pre- and Post-Intervention Trial

Each arm of the trial was conducted over a three-day period. The three-day span allowed for differing weather conditions and inter-day exposures. The HVAC system operated 24 hrs per day, and periods between 7 a.m. and 7 p.m. were classified as work periods whereas those between 7 p.m. and 7 a.m. were classified as night-time periods (unoccupied area).

The pre-intervention monitoring period was 9 August 2023 to 11 August 2023. During this period, the standard MERV-13 filter was in place. The MERV-13 filter has a 50–75% particle collection efficiency for 0.3–1.0 μm particles, >90% for 1.0–3.0 μm particles, and >90% for particles measuring 3–10 microns [24,26].

**Figure 2:**
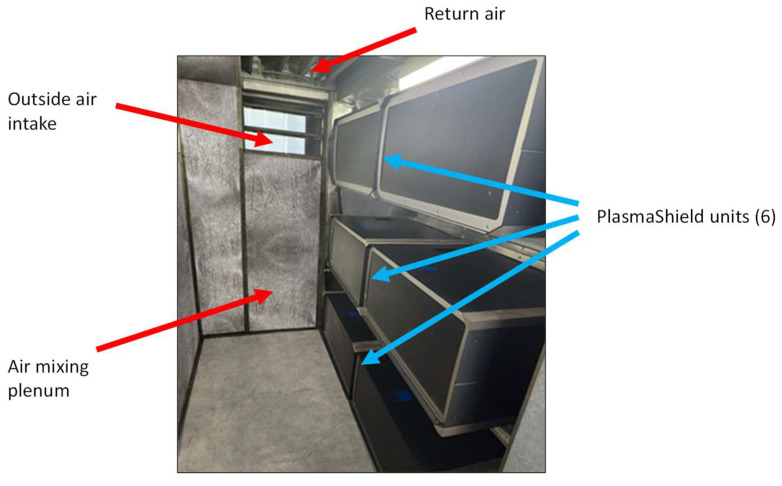
Six PlasmaShield units located in the air handling unit air mixing plenum.

The post-intervention monitoring period was 6 September 2023 to 8 September 2023. Here, a bank of six MMD-600 PlasmaShield units substituted for the existing MERV-13 filter that supplied air to the recovery suite (see Figure 2). There was no change in fan size or power, resulting in there being no energy gain or loss from the HVAC fan perspective.

### 2.3. Air Sampling

Continuous multi-size particle counting was conducted at the sampling points described above, along with assessment of other air quality parameters, including temperature, humidity, volatile organic compounds, formaldehyde and carbon dioxide (CO_2_). Supplementary measurements were also taken in the outside areas adjacent to the building housing the recovery suite, as well as in the recovery suite.

Calibrated instrumentation and standardised methods were used.

Periodic grab sampling of airborne culturable bacteria and fungi was also undertaken. One purpose of the grab sampling was to identify microbial species, complementing the generic airborne particle assessment.

#### 2.3.1. Sampling Methods

##### Air Quality Assessments (Non-Biological)

Airborne particle counts at one-minute intervals at the three locations were measured continuously and simultaneously using TSI AeroTrak handheld particle counters (Model 9306 v2, Shoreview MN, USA). Multiple locations were sampled to enable air quality comparisons.

Particle numbers in the range of 0.3–10 μm were measured, i.e., 0.3, 0.5, 1, 3, 5 and 10 μm. The particle number concentrations were the number of particles counted per cubic metre of air.

On a size basis, particles could be broadly classified as virus-like (VLP, 0.3–0.5 um), and bacteria-like (BLP 0.5–5 µm post hoc [27].

This size range fits well within the published size distributions of exhaled viruses and bacteria, as well as with aerosol measurements observed during vocalisation in an experimental setting [28].

A handheld TSI DustTrak DRX dust monitor was also used periodically as a complementary device at the various locations in the recovery suite and external to the building. The DustTrak particle values were consistent with the AeroTrak values.

Carbon dioxide, temperature, humidity and carbon monoxide (CO) were measured continuously using TSI Q-Track 7575 multifunction air quality monitors. Spot checks of nitrogen dioxide utilised a Aeroqual—Series 500 instrument (Auckland, New Zealand). Spot checks for ozone and sulphur dioxide utilised an InDevR 2B Technologies 202 instrument (Broomfield, CO, USA) and an Industrial Scientific MX6 Ibrid instrument (Pittsburgh, PA, USA), respectively.

VOCs and formaldehyde were sampled over 6–8 h periods using SKC Airchek XR5000 (Eighty Four, PA, USA) air sampling pumps with flow rates of 1 L/minute and 0.5 L/minute, respectively. Flow rates were checked with a Defender 510 calibrator (Mesa Labs, Lakewood, CO, USA). For VOCs, sampling was carried out using SKC sorbent tubes packed with coconut charcoal (SKC Anasorb CSC, 226-09). For formaldehyde, SKC Sorbent Tubes (226-119) containing high-purity silica gel treated with 2,4-dinitrophenyl hydrazine were employed.

VOCs and formaldehyde samples were analysed by TestSafe Australia using accredited standard methods.

Volumetric airflows through air supply registers were checked with a TSI 8380 Accubalance flow hood. Air movement (speed and directionality) in the occupied space was assessed using a Drager Cumulus air flow indicator (Lübeck, Germany). Real-time VOC assessment was undertaken with an Ion Science (Royston, UK) Tiger XT photoionisation detector (10.6 eV).

Surface temperatures were assessed with a Digitech QM 7226 (Electus Distribution, Rydalmere, NSW, Australia) non-contact infrared thermometer.

Simultaneous monitoring of environmental parameters such as CO_2_ concentration, temperature, humidity, and CO levels provided a holistic view of the indoor and outdoor air conditions and the level of occupancy during the study.

##### Microbial Assessments

Microbial measurements were undertaken by Bacterial and Mould Services, an expert microbial exposure assessment company. Air grab samples were taken for bacterial and fungal assessment, pre- and post-intervention, daily in the morning and in the afternoon, from the outside air, within the outside air intake, supply air and inside the recovery suite. Five-hundred-litre air samples were taken with a Spin Air (ThermoFisher Scientific, Waltham, MA, USA) sampler (IUL5532) to separate bacterial and fungal media.

Incubation was conducted under routine conditions (3 days for the bacterial load at 30–35 °C and 5 days for the fungal load at 26–28 °C). Basic identification and counting techniques were subsequently employed using optical microscopy.

Bacterial and fungal loads were expressed as colony-forming units per cubic metre (CFU/m^3^). For microbial assessment, bacterial and fungal loads were combined.

### 2.4. Statistics

Statistical assessment was mainly of a descriptive nature, stating summary statistics for particle number concentrations using the six pre-described size ranges by time of the day, pre- and post-intervention.

Box and whisker plots were used to describe the distribution of particle number concentrations for VLP and BLP.

Reduction in particle number concentrations (VLP and BLP) in the occupied recovery suite was expressed as the percent reduction from pre-intervention to post-intervention. The non-parametric Wilcoxon signed rank test was used to test the significance of differences, utilising Stata 18 software [29].

## 3. Results

### 3.1. General Observations

The recovery suite was essentially an open rectangular area measuring 220 m^2^ with a volume of approximately 660 m^3^. Air to the recovery suite was supplied at 4300 m^3^/h, giving a theoretical air exchange rate of 6.5 ACH of total air and 1.6 ACH based on outside air.

Assessment of air mixing with the smoke generator indicated reasonable air mixing, with air flowing from the recovery suite to the corridor adjacent to the operating theatres.

There were no obvious odours. However, staff were using disinfectant wipes and placing them into open bins, potentially contributing to VOC levels. This was confirmed by spot checks with the photoionisation detector, and also with the results of the 8 h sampling with charcoal tubes. The key VOCs were ethyl alcohol and isopropyl alcohol.

Parts per million levels of VOCs were recorded around the rubbish bins. Very low levels (<0.05 ppm) were recorded in outside areas and in the plant room.

Room temperature was around 25 °C and relative humidity ranged from 27 to 47%.

### 3.2. Airborne Particles

Raw graphical data for each of the six size channels (Figures S1–S12) and detailed statistics (Table S1) are provided in the Supplementary Materials, while tabulated summary data are given in Table 1 below.

Table 1 summarises the VLP- and the BLP-sized particle concentrations for outdoor air, supply air and the occupied space air, pre-and post-intervention, all day (total), during work shifts and during night-times, expressed as the mean, the standard deviation (SD) and the median number concentrations of particles per cubic metre.

During pre-intervention, the particle concentrations in the VLP category were reduced by 87% in the supply air and by 91% in the occupied room air compared to those in the outdoor air over the total time period. During the work period, the reduction was 84% in the supply air and 88% in the occupied room. At night, the comparative reduction was 88% in the supply air and 92% in the occupied room air.

After the intervention, the VLP values showed a reduction of 99.9% in the supply air compared to the outdoor air over the total time period. During workhours, the reduction was 99.9% in the supply air and 97% in the occupied room air. During the night, the reduction in the supply air was >99.9%, and in the occupied room, it was 99%.

The BLP values showed no difference between outdoor air and supply air pre-intervention for any of the time categories. Differences were seen between outdoor and occupied room air when considering the total time period (27%), during workhours (9.9%) and at night (35%).

The intervention-related reduction in the BLP category compared with the outdoor air was observed for the total time period in the supply air (99.9%) and the occupied room air (98.5%), and during workhours in the supply air (>99.9%) and in the occupied room air (97%). At night, reductions were observed in the supply air (>99.9%) and the occupied room air (99.4%).

The reduction in mean total particle number concentrations between the occupied room air before and after the intervention was high for the channels measuring 0.3 μm (>14 fold), 0.5 μm (6-fold) and 1 μm (>2 fold). Using the mean values, the decrease in VLP in the occupied space post-intervention compared to pre-intervention during work shifts was 8-fold and for BLP it was 2.7-fold.

For the particle sizes 3–10 μm, no further major reductions were achieved, indicating that PlasmaShield, compared with MERV-13, is mostly relevant for reducing VLP and BLP particles.

**Figure 3:**
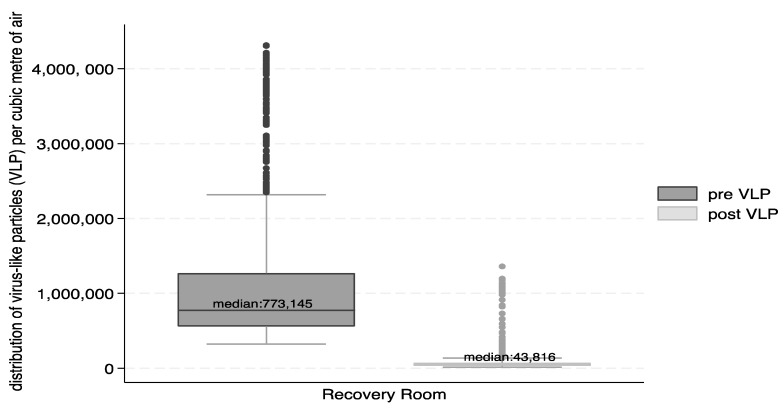
Box and whisker plot of the distribution of VLP particles (count per m^3^) in the recovery room pre- and post-intervention.

**Figure 4:**
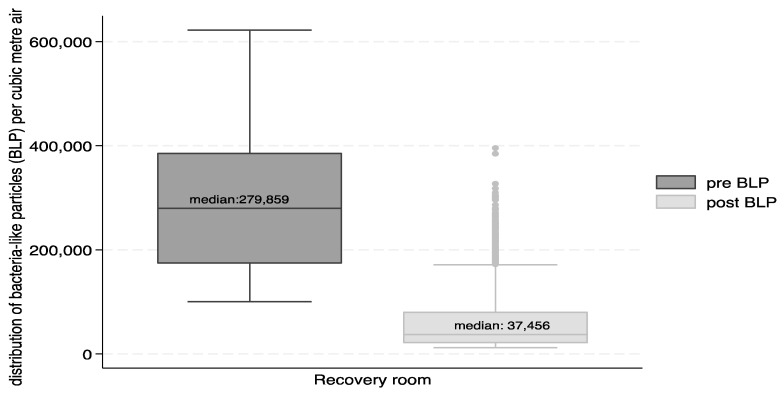
Box and whisker plot of the distribution of BLP-sized particles (count per m^3^) in the recovery room pre- and post-intervention.

Figure 3 and Figure 4 show the reductions in the form of box and whisker plots. The median number of VLP particles per cubic metre of air was reduced from 773,145 to 43,816, and that for BLP was reduced from 279,859 to 37,456.

The percent reduction in the total number of particles in the recovery suite between the pre- and post-intervention was 94% for VLP and 87% for BLP. The Wilcoxon signed rank test indicated that the differences were significant (*p* < 0.0001) for total VLP and BLP, as well as for VLP and BLP differences in the recovery suite during the work shifts and during night-time.

### 3.3. Culturable Microbial Counts

For all grab samples, the types of bacteria and fungi cultured were common environmental saprophytes, which are widespread throughout many environments and can be readily disseminated by ventilation systems and indoor occupants.

Compared with outdoor air, significant reductions in culturable bacteria and fungi were observed at the exit point of the PlasmaShield unit.

From Table 2, it can be seen that in the case of bacteria, the PlasmaShield unit was able to reduce the total microbial load by 98% compared to only 41% with the original MERV-13.

In the case of fungi, the PlasmaShield unit reduced the total microbial load by 96% compared to 90% with the original MERV-13.

Overall, the PlasmaShield unit reduced the total microbial load by 96% compared to 71% in the original MERV-13.

When comparing PlasmaShield with the original MERV-13 filter, further reductions in culturable bacteria and fungi were observed in the occupied space, but these were modest and variable and can be attributed to the human occupancy and activity, as described in the Discussion.

### 3.4. VOCs, Formaldehyde, Ozone, Nitrogen Dioxide, Sulphur Dioxide, Carbon Monoxide and Carbon Dioxide

Volatile organic compound and formaldehyde concentrations were low, and the biggest contributions probably arose from the regular use of cleaning agents and disinfectants, as well as from off-gassing from indoor surfaces.

#### 3.4.1. Volatile Organic Compounds

VOC concentrations were low/there was a slight reduction in VOCs post-intervention in the recovery suite. The values were as follows: pre-intervention, 1282 µg/m^3^ (n = 5); post-intervention, 1046 µg/m^3^ (n = 5).

The VOCs were predominantly ethyl alcohol and isopropyl alcohol, derived from the use of disinfecting agents and the work practice of disposing disinfectant materials in open bins.

#### 3.4.2. Formaldehyde

The measured values were low, below the odour threshold of approximately 50 µg/m^3^.

The values were as follows: pre-intervention, 7.6 µg/m^3^ (n = 3); post-intervention, 8.3 µg/m^3^ (n = 3).

The World Health Organization (WHO) IAQ guideline for formaldehyde is 100 µg/m^3^ [30].

#### 3.4.3. Ozone

Ozone levels in the recovery suite were below the limit of detection (<1 ppb).

#### 3.4.4. Nitrogen Dioxide, Sulphur Dioxide and Carbon Monoxide

The levels were low and below accepted standards. Nitrogen dioxide levels were typically less than 10 ppb (maximum was 30 ppb). Carbon monoxide and sulphur dioxide levels were generally too low for reliable detection (<1 ppm and 0.1 ppm).

#### 3.4.5. Carbon Dioxide

These were variable in the normal range and similar pre- and post-intervention (400–750 ppm).

As the predominant source of carbon dioxide is humans, the data indicated that the occupancy was similar both pre- and post-intervention.

## 4. Discussion

The PlasmaShield system is a new technology. Unlike UV/HEPA systems, it uses electron beams and electric fields to inactivate bioaerosols, which also serve to aggregate very small particles so that they can be captured efficiently by filters that do not suffer from the large pressure drops associated with HEPA [24]. This is the first study of such a device in a real-life setting, in this case a post-surgical paediatric clinical setting. As such, it adds to the existing evidence base on advanced air treatment systems that address the triple challenge of improved IAQ, infectious disease reduction and energy efficiency.

This trial was not able to clearly demonstrate reductions in VOCs and gases in the occupied space, as these were at very low levels, below accepted IAQ standards and odour thresholds. The activity and use of chemicals in the occupied space generated VOCs and bioaerosols.

There are a number of limitations to this study.

PlasmaShield lifespan considerations such as comparative air quality, power consumption and economic factors could not be assessed in the three-day observation period. Long-term studies are under way. Seasonal variability in air quality was not assessed. However, work schedules, practice and occupancy should not be significantly different. Surface sampling was not conducted, and it was not feasible to attach personal air sampling devices to nursing staff and monitor for a shift. Viruses were not directly measured, and “grab” sampling for culturable airborne bacteria and fungi could only be conducted twice per day when there was reduced activity and occupancy. This is therefore a gross measure and unlikely to be representative of the average microbial load in the air.

Mitigating the limitations above, continuous real-time multi-size particle counting was carried out, providing rich and representative data, but without microbial speciation. Before and after measurements, in the same environmental conditions as the particle counting instrumentation was a robust reflection of the effectiveness of the intervention, which has been used in multiple studies of air cleaning systems [22,24].

Evidence of the transmission of respiratory viral disease in the form of aerosols has been widely acknowledged [4,5,7]. It has been recognised that virus-containing aerosols can be ejected during respiratory exhalations from proximal (close-range, ballistic) to far distances (>2 m). Depending on the activities, particle number concentrations change with a significant difference between breathing and vocalisation, as well as based on the sound volume measured in decibels (dB) [28]. Moreover, in an experimental study with children and adults, vocalisation seems to produce a bimodal aerosol size distribution with the size of the smaller particles being 0.50–0.64 μm in diameter, while the larger particles were around 1.39–1.94 μm [28]. Aerosol collection experiments with COVID-19 patients found that SARS-CoV-2 RNA during vocalisation spanned 0.34->8.1 μm with 90% of the particles being <4.5 μm [31]. The sub-micron range of particle virus ejection due to vocalisation has been supported elsewhere [32]. This evidence, plus research on outdoor air, suggests that a focus in achieving indoor clean air should be on the sub-micron particles [33].

In hindsight of the COVID-19 pandemic, and in the absence of any relevant guidance, the American Society of Heating, Refrigerating and Air Conditioning Engineers (ASHRAE) recently developed clean air indoor standards that are relevant for infection risk [34].

**Figure 5:**
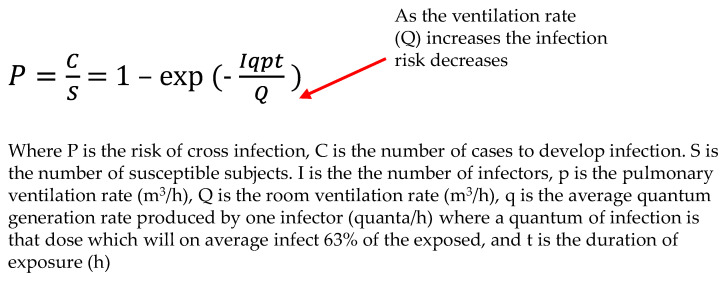
The Wells–Riley equation, highlighting the infection risk relationship with the room ventilation rate.

The Wells–Riley equation, as shown in Figure 5, can be used to predict the relative infection risk reduction in this study [23].

To evaluate the effectiveness of control measures, the eight-fold-reduced VLP (886,302 vs. 108,630 particles) particle number concentration post-intervention compared to that pre-intervention during work shifts was assessed via the Wells–Riley equation. Thus, it is inferred that the eight-fold post-intervention reduction in VLP serves as the equivalent to an eight-fold increased ventilation rate (m^3^/h) in clean air. Adding the increased ventilation rate into the Wells–Riley formula, P = 1 − e ^(−1/8)^, results in a 5.33-fold risk reduction. This calculation provides an example of a solution for equivalent clean airflow through air cleaning devices, as described in the new ASHRAE standard [34]. In the case of BLP, there is a 2.7-fold concentration reduction, which translates to about a 2-fold risk reduction.

HEPA filters are widely used in critical environments, according more than 99% protection from sub-micron particles. However, as shown in a hypothetical office environment study, HEPA filters compared to MERV filters are costly due to the high energy requirements for overcoming airflow restrictions [35]. Here, the annual fan energy cost for HEPA was more than twice as high as for MERV-13. The issue of unforeseen environmental indoor aerosol production associated with the UV-C ionising lamps has also recently been raised [36].

This study was not designed to demonstrate reductions in infectious health effects in patients, visitors or staff. In future, health outcome improvements observed from a similar clinical setting or from a GP clinic would provide further validation of PlasmaShield. This is especially pertinent considering that there are an estimated 165,000 healthcare-associated infections in Australia per year, many of which are preventable [37]. Furthermore, sub-micron airborne particles derived from general air pollution or major events, such as from wildfires, could be captured and removed from airstreams by this device, which has applicability beyond the clinical care space. Further studies in buildings are needed to evaluate the health and productivity benefits for occupants.

## 5. Conclusions

An advanced multi-modal air purification system was retrofitted into a MERV-13 filter-based HVAC system of a post-surgical hospital recovery suite, and the resultant aerosol reduction was HEPA-like in performance. Using continuous particle number counting across six size ranges in three air sampling locations, the most noteworthy reduction occurred for submicron particles, where a greater-than-eight-fold reduction was seen in the occupied space during work hours. A relative health risk reduction is inferred. A five-fold infection risk reduction is estimated for airborne viruses in a long-range exposure scenario. There were no changes made to fan size or power to achieve the observed particle reduction performance. In addition, the much less contaminated air returning from the occupied space had the potential to reduce the outside air requirement and to reduce energy costs. Long-term studies are recommended to confirm the combined IAQ, health and energy benefits.

## Figures and Tables

**Table 1:** Pre- and post-intervention VLP and BLP concentrations (per cubic metre) for outdoor air, supply air and occupied space air. Values (mean, standard deviation and median) are divided by 10^3^ and relate to total hours, work hours and night-time.

	Pre-Intervention (MERV-13)							Post-Intervention (PlasmaShield)					
	VLP			BLP				VLP			BLP		
No. of Samples	Mean	SD	Median	Mean	SD	Median	No. of Samples	Mean	SD	Median	Mean	SD	Median
Outdoor air													
**Total** 2757	11,802	11,079	5682	389	591	358	2961	3562	1549	3662	4074	2429	4253
**Work** 1328	7194	5733	4279	284	1588	253	1526	3251	1093	3571	3633	1533	4139
**Night** 1429	15,907	12,956	12,101	473	2155	451	1435	3891	1861	3740	4540	3038	4674
Supply Air													
**Total** 2757	1554	1205	964	389	179	358	2961	2	3	1	1	1	0.7
**Work** 1328	1162	702	826	284	135	253	1526	3	4	2	1	1	1
**Night** 1429	1867	1504	1544	473	165	451	1435	1	0.7	0.7	0.8	0.5	0.7
Room Air													
**Total** 2757	1060	713	773	282	116	279	2961	74	112	43	61	59	37
**Work** 1328	886	469	724	256	112	240	1526	108	149	67	94	67	79
**Night** 1429	1216	899	1013	306	115	296	1435	38	14	36	27	13	21

**Table 2:** Bacterial and fungal reduction in the supply air compared to the outdoor air, pre- and post-intervention.

	Pre-Intervention (CFU/m^3^)	Post-Intervention (CFU/m^3^)
**Bacteria**		
Outdoor air	202	166
Supply air	120	4
Reduction (%)	41%	98%
**Fungi**		
Outdoor air	314	320
Supply air	30	14
Reduction (%)	90%	96%

## Data Availability

The raw dataset supporting the conclusions will be available on request from the authors.

## Supplementary Materials

The following supporting information can be downloaded at https://www.mdpi.com/article/10.3390/ijerph21081089/s1: Figures S1–S12: caption: The 12 time series figures describe the pre-and post-intervention particle number concentrations (/m^3^ air) determined via the measurement channels for particles measuring 0.3, 0.5, 1, 3, 5 and 10 µm. Work periods are indicated. Table S1: Mean and standard deviation (SD) of particles by channel size (0.3, 0.5, 1, 3, 5 and 10 µm) pre- and post-intervention for outdoor, supply and recovery room air, summarised as those for the total time, during work hours and at night.
