# Supplementary material for: Relative Health Risk Reduction from an Advanced Multi-Modal Air Purification System: Evaluation in a Post-Surgical Healthcare Setting
Source: Int J Environ Res Public Health. 2024 Aug 17;21(8):1089. doi: 10.3390/ijerph21081089 (PMC11354897; doi:10.3390/ijerph21081089)
Supplement: Supplementary file 1 [file ijerph-21-01089-s001.zip › ijerph-3051640-supplementary.pdf]

Supplementary Material

Relative health risk reduction from an advanced multi-modal air purification system: Evaluation in a post-surgical healthcare setting

Authors: Pisaniello D, Nitschke M

Figures S1–12: The 12 time series graphs describe the pre-and post-intervention particle number concentrations for 0.3, 0.5, 1, 3, 5 and 10 µm size ranges. Work periods are indicated.

The progression of particle number concentrations during the pre and post intervention periods is demonstrated in twelve time series graphs for the six channel sizes including 0.3µm, 0.5µm, 1µm, 3µm, 5µm and 10µm, for outdoor air intake, supply air, and the air in the recovery suite indicating work shifts and night-time. The graphs indicate reductions in particles in the supply and the occupied room air compared to outdoor air for both pre-and post-intervention. The reductions are substantially increased post intervention for fine particulate matter ranging from 0.3µm sized channels and 0.5µm. The supply air particle concentration is reduced to almost zero from the 1µm channel onwards post intervention, with a few spikes during work shifts. Night-time particle concentrations of the sizes 5-10µm in the recovery suite veered towards zero for pre and post intervention.

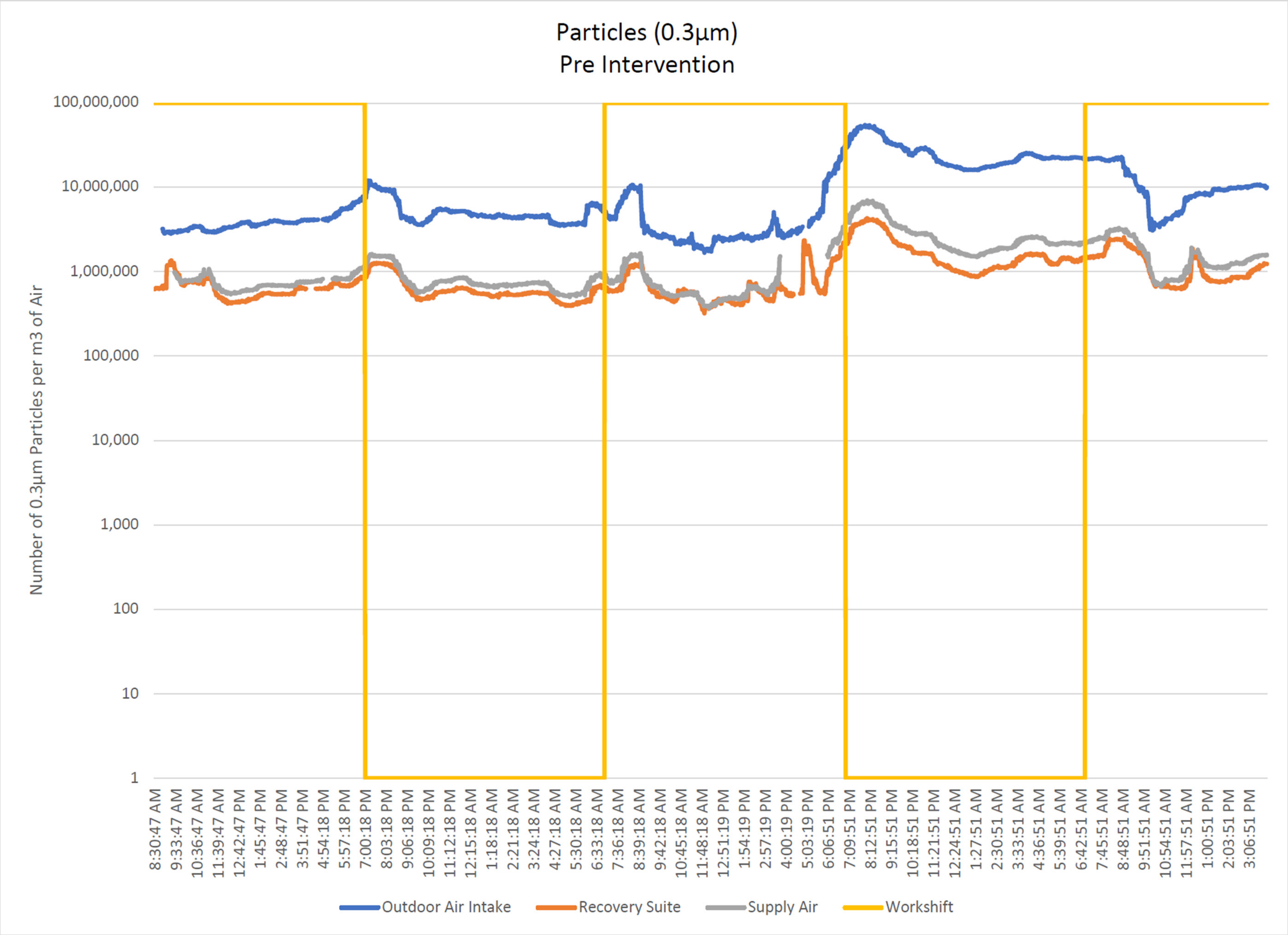
Particles (0.3µm)
Pre Intervention
Number of 0.3µm Particles per m3 of Air
100,000,000
10,000,000
1,000,000
100,000
10,000
1,000
100
10
1
8:30:47 AM
9:33:47 AM
10:36:47 AM
11:39:47 AM
12:42:47 PM
1:45:47 PM
2:48:47 PM
3:51:47 PM
4:54:18 PM
5:57:18 PM
7:00:18 PM
8:03:18 PM
9:06:18 PM
10:09:18 PM
11:12:18 PM
12:15:18 AM
1:18:18 AM
2:21:18 AM
3:24:18 AM
4:27:18 AM
5:30:18 AM
6:33:18 AM
7:36:18 AM
8:39:18 AM
9:42:18 AM
10:45:18 AM
11:48:18 AM
12:51:19 PM
1:54:19 PM
2:57:19 PM
4:00:19 PM
5:03:19 PM
6:06:51 PM
7:09:51 PM
8:12:51 PM
9:15:51 PM
10:18:51 PM
11:21:51 PM
12:24:51 AM
1:27:51 AM
2:30:51 AM
3:33:51 AM
4:36:51 AM
5:39:51 AM
6:42:51 AM
7:45:51 AM
8:48:51 AM
9:51:51 AM
10:54:51 AM
11:57:51 AM
1:00:51 PM
2:03:51 PM
3:06:51 PM
Outdoor Air Intake
Recovery Suite
Supply Air
Workshift

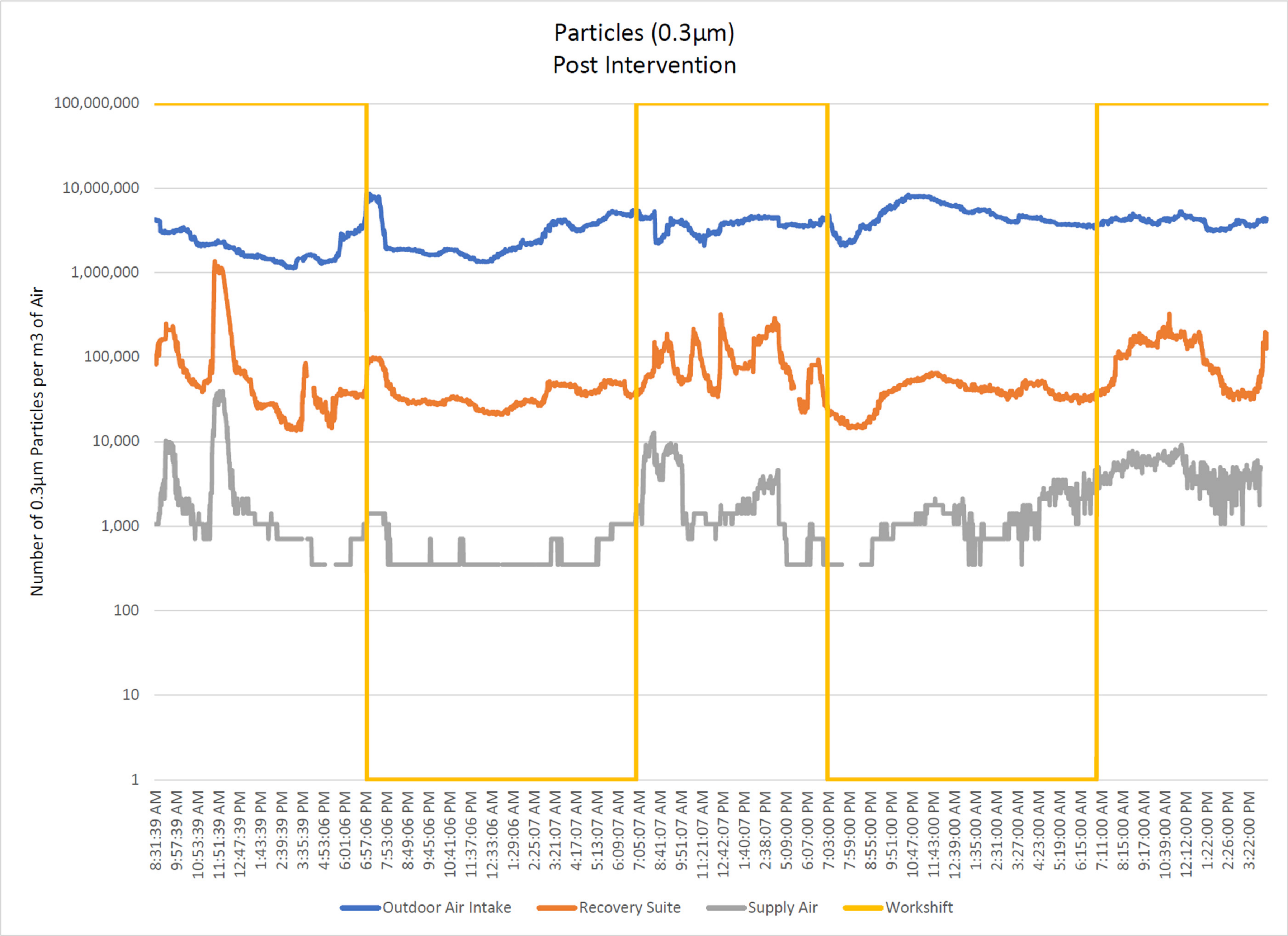
Particles (0.3µm)
Post Intervention
Number of 0.3µm Particles per m3 of Air
100,000,000
10,000,000
1,000,000
100,000
10,000
1,000
100
10
1
8:31:39 AM
9:57:39 AM
10:53:39 AM
11:51:39 AM
12:47:39 PM
1:43:39 PM
2:39:39 PM
3:35:39 PM
4:53:06 PM
6:01:06 PM
6:57:06 PM
7:53:06 PM
8:49:06 PM
9:45:06 PM
10:41:06 PM
11:37:06 PM
12:33:06 AM
1:29:06 AM
2:25:07 AM
3:21:07 AM
4:17:07 AM
5:13:07 AM
6:09:07 AM
7:05:07 AM
8:41:07 AM
9:51:07 AM
11:21:07 AM
12:42:07 PM
1:40:07 PM
2:38:07 PM
5:09:00 PM
6:07:00 PM
7:03:00 PM
7:59:00 PM
8:55:00 PM
9:51:00 PM
10:47:00 PM
11:43:00 PM
12:39:00 AM
1:35:00 AM
2:31:00 AM
3:27:00 AM
4:23:00 AM
5:19:00 AM
6:15:00 AM
7:11:00 AM
8:15:00 AM
9:17:00 AM
10:39:00 AM
12:12:00 PM
1:22:00 PM
2:26:00 PM
3:22:00 PM
Outdoor Air Intake
Recovery Suite
Supply Air
Workshift

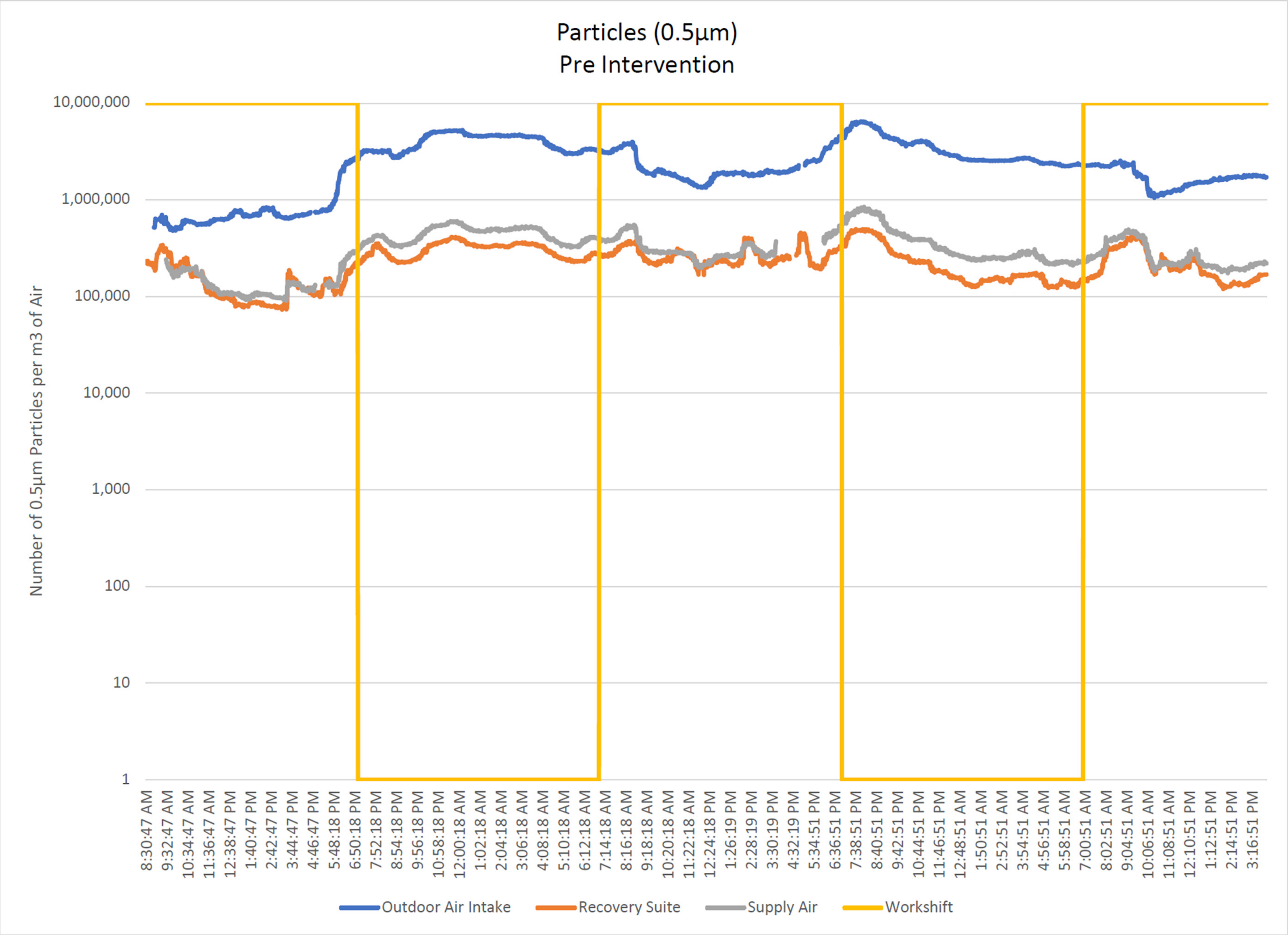

Particles (0.5µm)
Pre Intervention
Number of 0.5µm Particles per m3 of Air
10,000,000
1,000,000
100,000
10,000
1,000
100
10
1
8:30:47 AM
9:32:47 AM
10:34:47 AM
11:36:47 AM
12:38:47 PM
1:40:47 PM
2:42:47 PM
3:44:47 PM
4:46:47 PM
5:48:18 PM
6:50:18 PM
7:52:18 PM
8:54:18 PM
9:56:18 PM
10:58:18 PM
12:00:18 AM
1:02:18 AM
2:04:18 AM
3:06:18 AM
4:08:18 AM
5:10:18 AM
6:12:18 AM
7:14:18 AM
8:16:18 AM
9:18:18 AM
10:20:18 AM
11:22:18 AM
12:24:18 PM
1:26:19 PM
2:28:19 PM
3:30:19 PM
4:32:19 PM
5:34:51 PM
6:36:51 PM
7:38:51 PM
8:40:51 PM
9:42:51 PM
10:44:51 PM
11:46:51 PM
12:48:51 AM
1:50:51 AM
2:52:51 AM
3:54:51 AM
4:56:51 AM
5:58:51 AM
7:00:51 AM
8:02:51 AM
9:04:51 AM
10:06:51 AM
11:08:51 AM
12:10:51 PM
1:12:51 PM
2:14:51 PM
3:16:51 PM
Outdoor Air Intake
Recovery Suite
Supply Air
Workshift

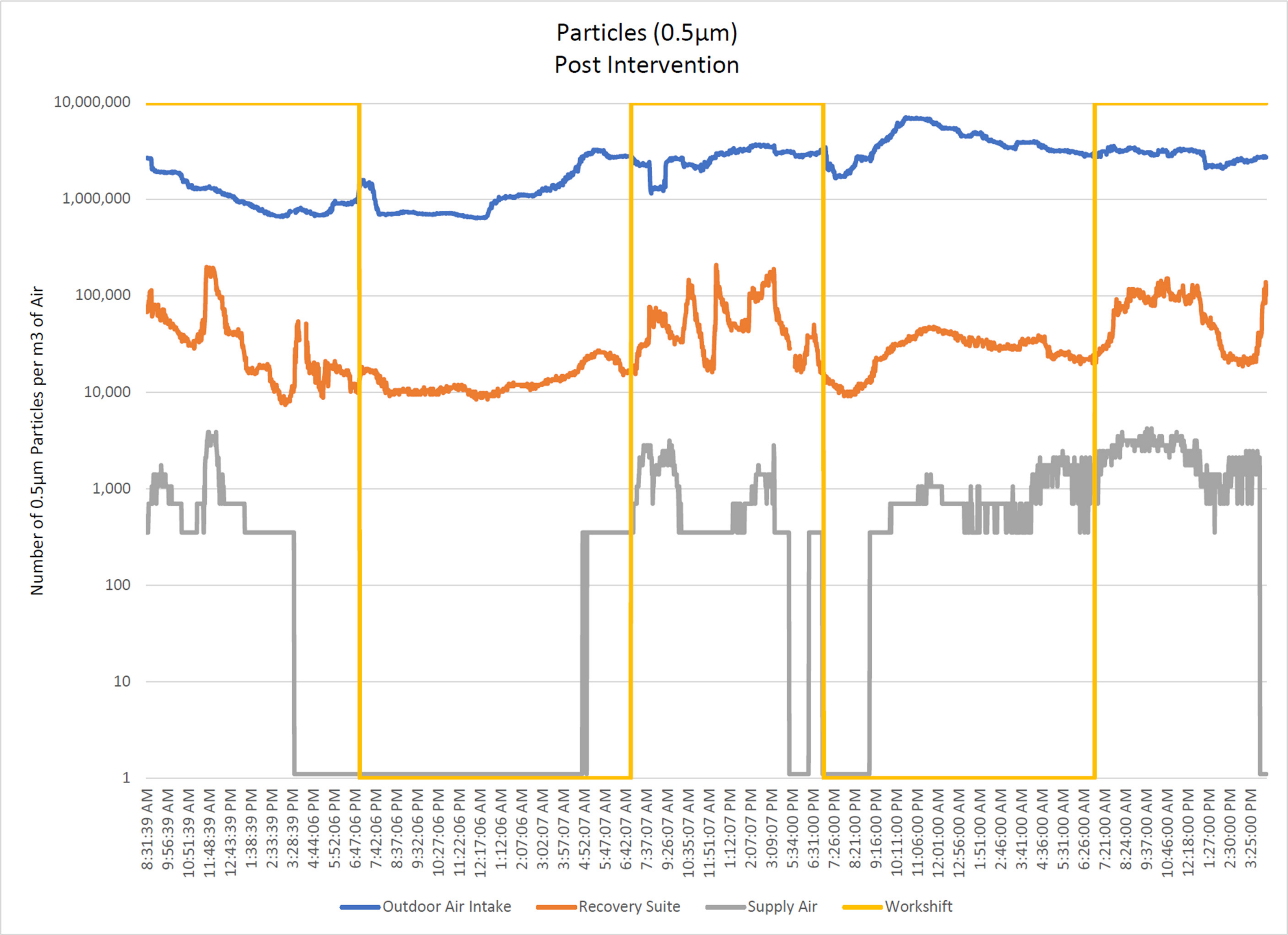

Particles (0.5µm)
Post Intervention
Number of 0.5µm Particles per m3 of Air
10,000,000
1,000,000
100,000
10,000
1,000
100
10
1
8:31:39 AM
9:56:39 AM
10:51:39 AM
11:48:39 AM
12:43:39 PM
1:38:39 PM
2:33:39 PM
3:28:39 PM
4:44:06 PM
5:52:06 PM
6:47:06 PM
7:42:06 PM
8:37:06 PM
9:32:06 PM
10:27:06 PM
11:22:06 PM
12:17:06 AM
1:12:06 AM
2:07:06 AM
3:02:07 AM
3:57:07 AM
4:52:07 AM
5:47:07 AM
6:42:07 AM
7:37:07 AM
9:26:07 AM
10:35:07 AM
11:51:07 AM
1:12:07 PM
2:07:07 PM
3:09:07 PM
5:34:00 PM
6:31:00 PM
7:26:00 PM
8:21:00 PM
9:16:00 PM
10:11:00 PM
11:06:00 PM
12:01:00 AM
12:56:00 AM
1:51:00 AM
2:46:00 AM
3:41:00 AM
4:36:00 AM
5:31:00 AM
6:26:00 AM
7:21:00 AM
8:24:00 AM
9:37:00 AM
10:46:00 AM
12:18:00 PM
1:27:00 PM
2:30:00 PM
3:25:00 PM
Outdoor Air Intake
Recovery Suite
Supply Air
Workshift

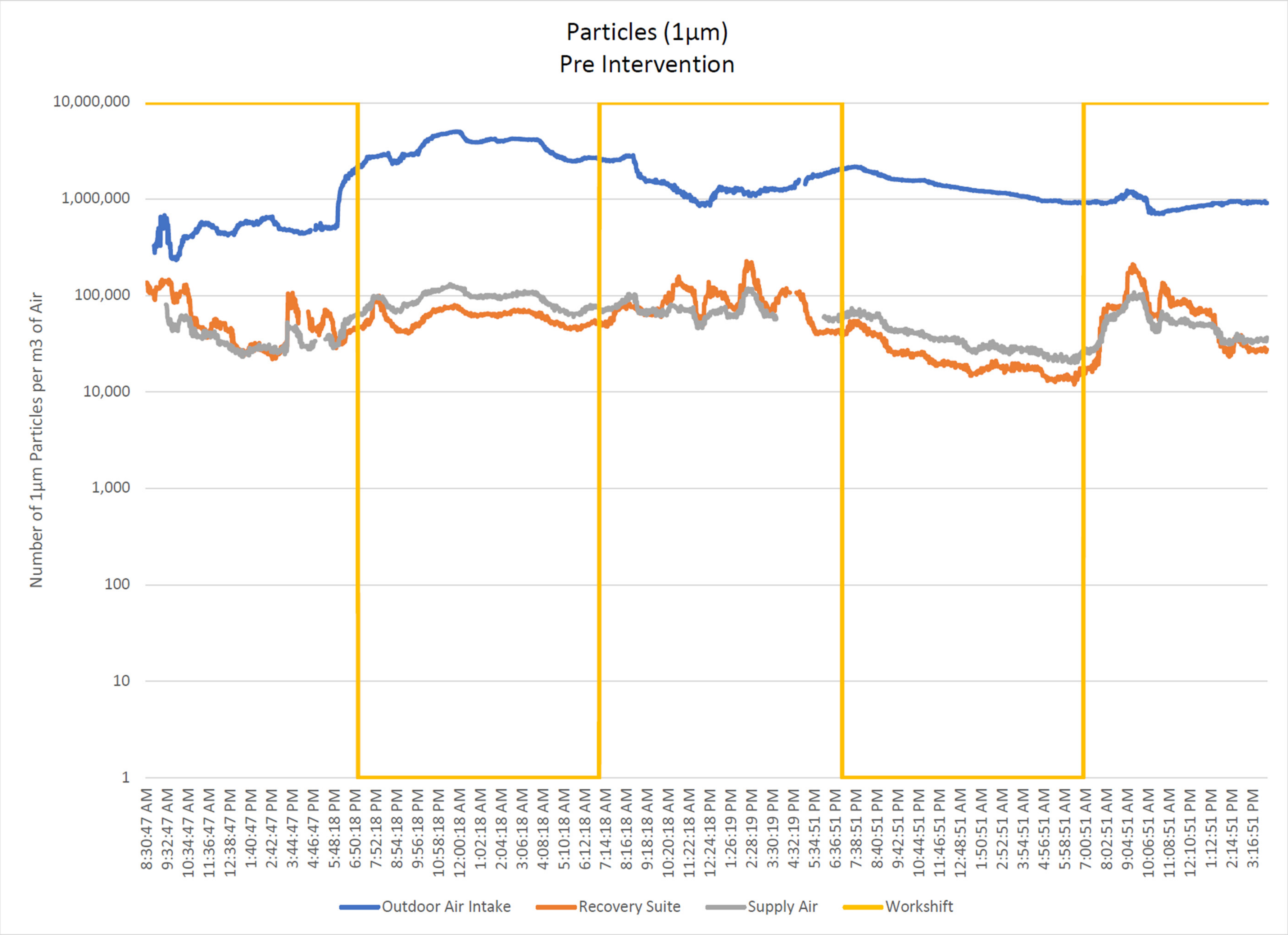

Particles (1µm)
Pre Intervention
10,000,000
1,000,000
100,000
10,000
1,000
100
10
1
Number of 1µm Particles per m3 of Air
8:30:47 AM
9:32:47 AM
10:34:47 AM
11:36:47 AM
12:38:47 PM
1:40:47 PM
2:42:47 PM
3:44:47 PM
4:46:47 PM
5:48:18 PM
6:50:18 PM
7:52:18 PM
8:54:18 PM
9:56:18 PM
10:58:18 PM
12:00:18 AM
1:02:18 AM
2:04:18 AM
3:06:18 AM
4:08:18 AM
5:10:18 AM
6:12:18 AM
7:14:18 AM
8:16:18 AM
9:18:18 AM
10:20:18 AM
11:22:18 AM
12:24:18 PM
1:26:19 PM
2:28:19 PM
3:30:19 PM
4:32:19 PM
5:34:51 PM
6:36:51 PM
7:38:51 PM
8:40:51 PM
9:42:51 PM
10:44:51 PM
11:46:51 PM
12:48:51 AM
1:50:51 AM
2:52:51 AM
3:54:51 AM
4:56:51 AM
5:58:51 AM
7:00:51 AM
8:02:51 AM
9:04:51 AM
10:06:51 AM
11:08:51 AM
12:10:51 PM
1:12:51 PM
2:14:51 PM
3:16:51 PM
Outdoor Air Intake
Recovery Suite
Supply Air
Workshift

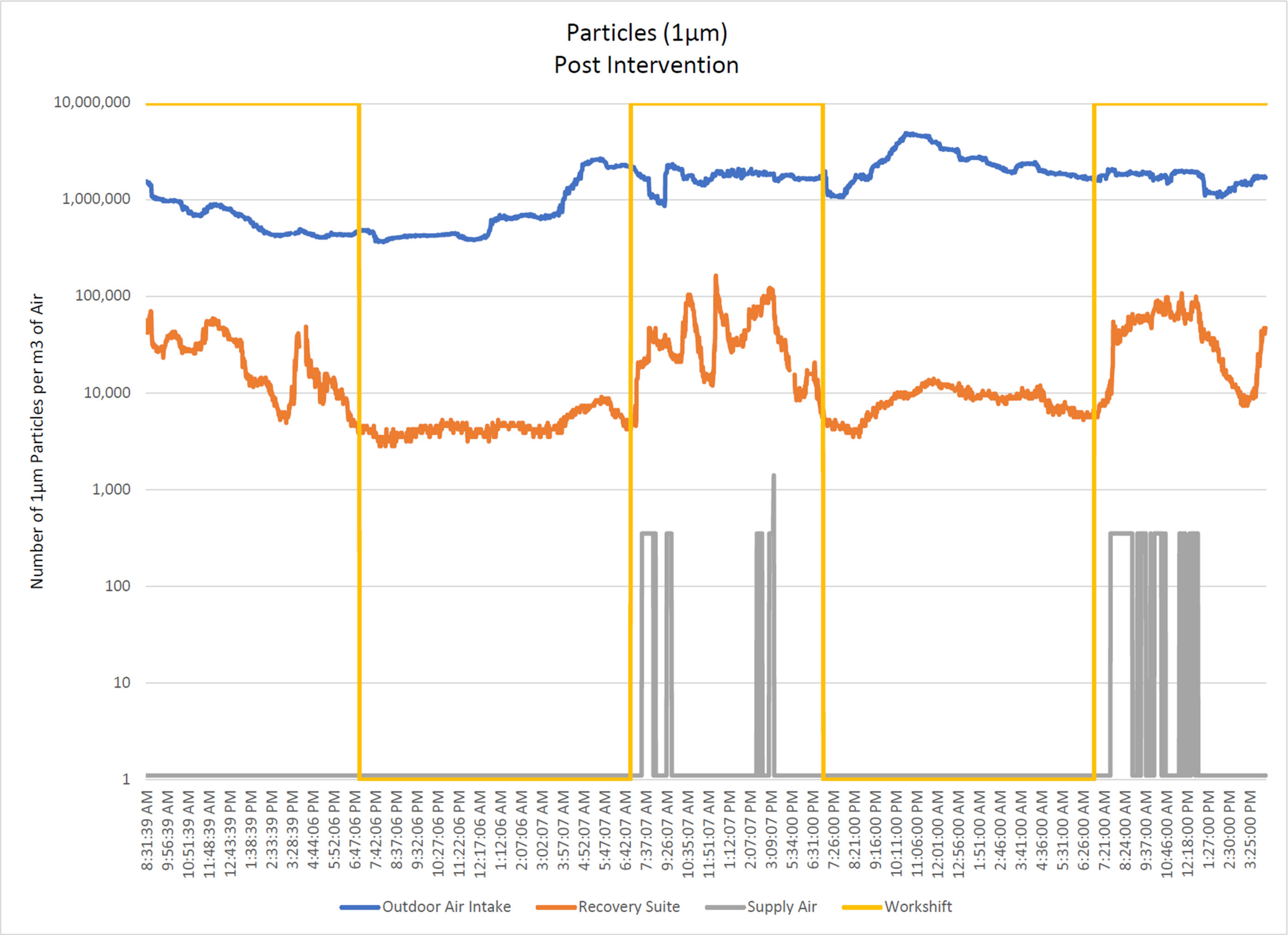

Particles (1µm)
Post Intervention
Number of 1µm Particles per m3 of Air
10,000,000
1,000,000
100,000
10,000
1,000
100
10
1
8:31:39 AM
9:56:39 AM
10:51:39 AM
11:48:39 AM
12:43:39 PM
1:38:39 PM
2:33:39 PM
3:28:39 PM
4:44:06 PM
5:52:06 PM
6:47:06 PM
7:42:06 PM
8:37:06 PM
9:32:06 PM
10:27:06 PM
11:22:06 PM
12:17:06 AM
1:12:06 AM
2:07:06 AM
3:02:07 AM
3:57:07 AM
4:52:07 AM
5:47:07 AM
6:42:07 AM
7:37:07 AM
9:26:07 AM
10:35:07 AM
11:51:07 AM
1:12:07 PM
2:07:07 PM
3:09:07 PM
5:34:00 PM
6:31:00 PM
7:26:00 PM
8:21:00 PM
9:16:00 PM
10:11:00 PM
11:06:00 PM
12:01:00 AM
12:56:00 AM
1:51:00 AM
2:46:00 AM
3:41:00 AM
4:36:00 AM
5:31:00 AM
6:26:00 AM
7:21:00 AM
8:24:00 AM
9:37:00 AM
10:46:00 AM
12:18:00 PM
1:27:00 PM
2:30:00 PM
3:25:00 PM
Outdoor Air Intake
Recovery Suite
Supply Air
Workshift

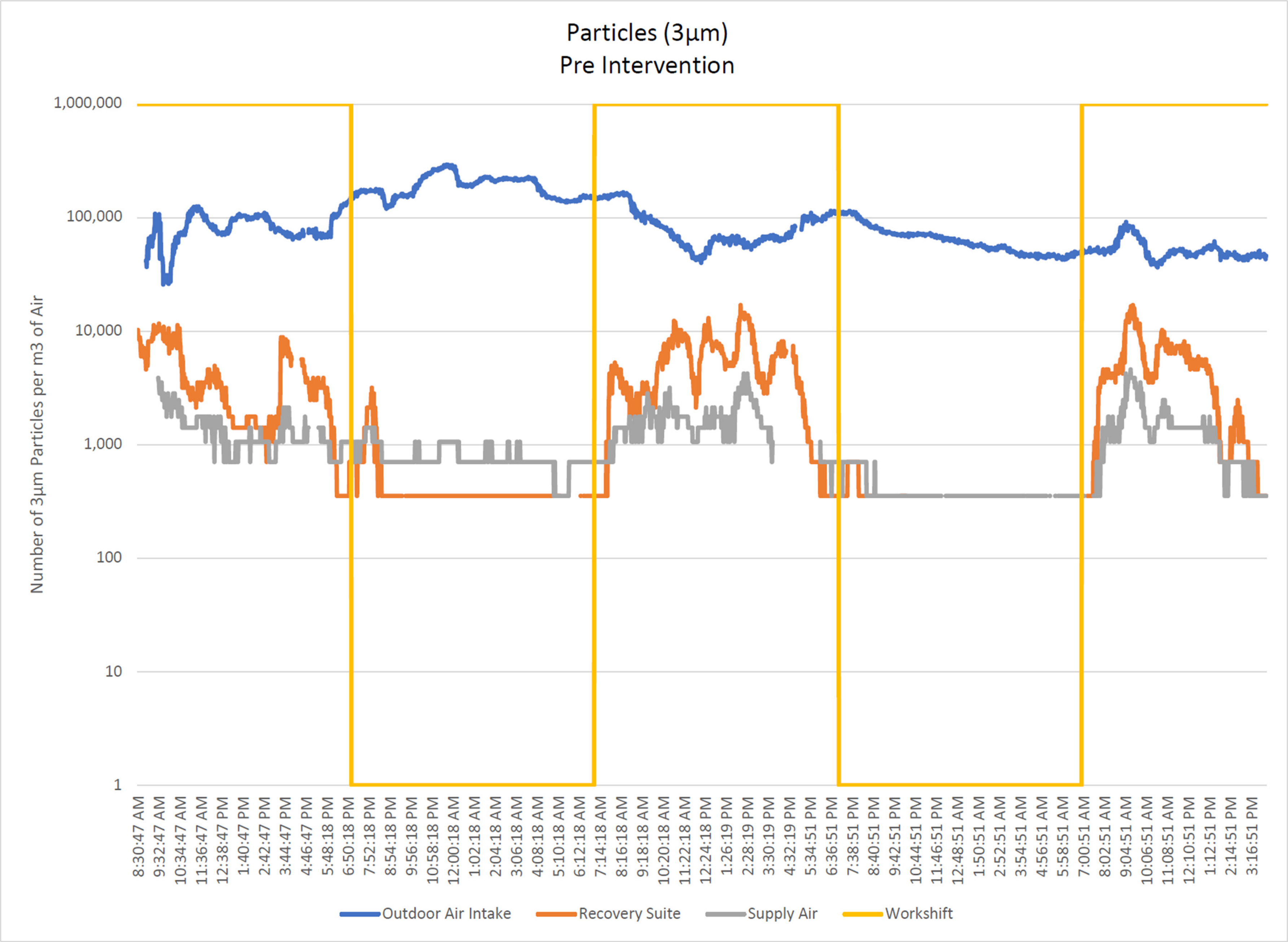
Particles (3µm)
Pre Intervention
Number of 3µm Particles per m3 of Air
1,000,000
100,000
10,000
1,000
100
10
1
8:30:47 AM
9:32:47 AM
10:34:47 AM
11:36:47 AM
12:38:47 PM
1:40:47 PM
2:42:47 PM
3:44:47 PM
4:46:47 PM
5:48:18 PM
6:50:18 PM
7:52:18 PM
8:54:18 PM
9:56:18 PM
10:58:18 PM
12:00:18 AM
1:02:18 AM
2:04:18 AM
3:06:18 AM
4:08:18 AM
5:10:18 AM
6:12:18 AM
7:14:18 AM
8:16:18 AM
9:18:18 AM
10:20:18 AM
11:22:18 AM
12:24:18 PM
1:26:19 PM
2:28:19 PM
3:30:19 PM
4:32:19 PM
5:34:51 PM
6:36:51 PM
7:38:51 PM
8:40:51 PM
9:42:51 PM
10:44:51 PM
11:46:51 PM
12:48:51 AM
1:50:51 AM
2:52:51 AM
3:54:51 AM
4:56:51 AM
5:58:51 AM
7:00:51 AM
8:02:51 AM
9:04:51 AM
10:06:51 AM
11:08:51 AM
12:10:51 PM
1:12:51 PM
2:14:51 PM
3:16:51 PM
Outdoor Air Intake
Recovery Suite
Supply Air
Workshift

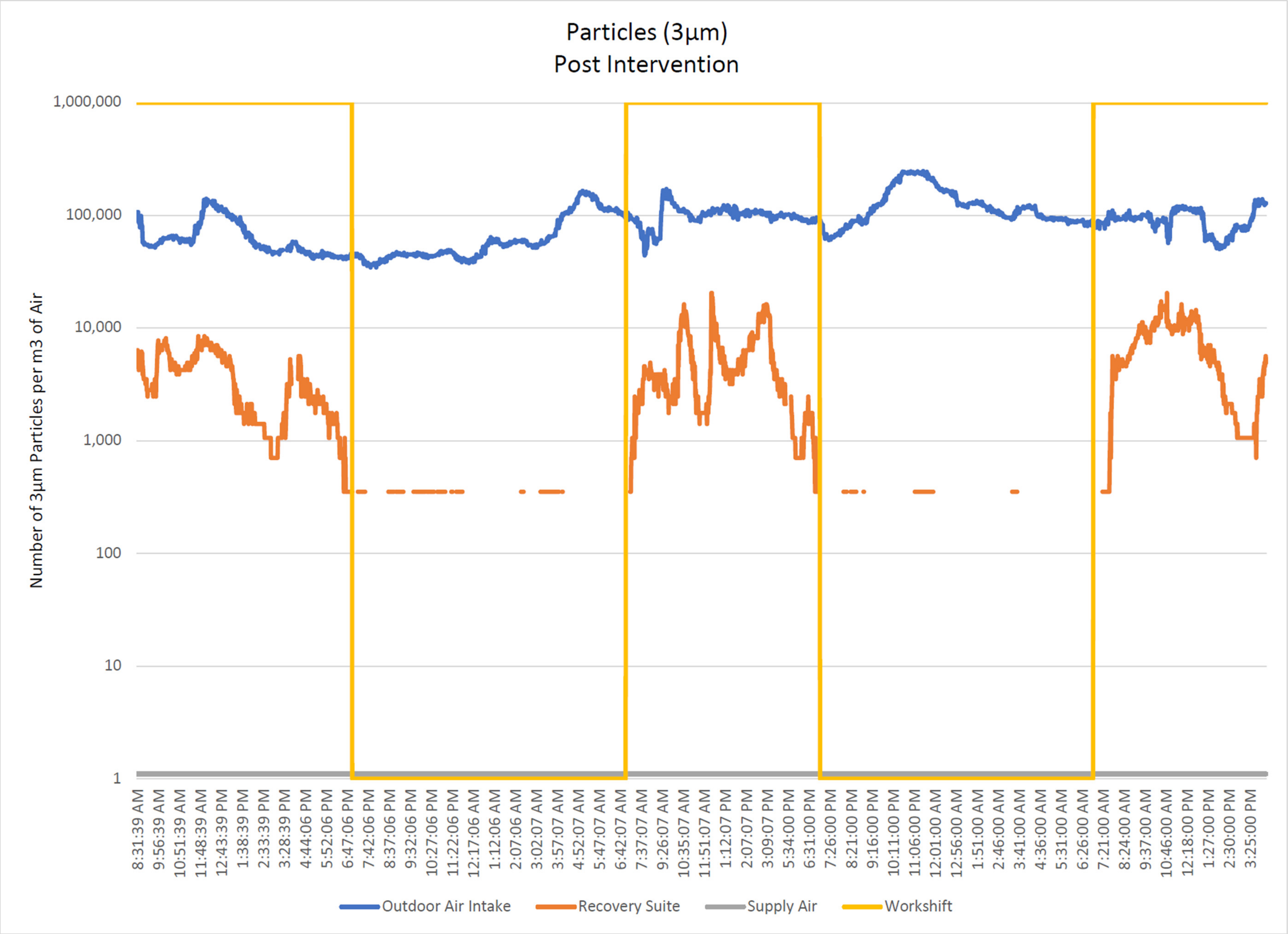

Particles (3µm)
Post Intervention
Number of 3µm Particles per m3 of Air
1,000,000
100,000
10,000
1,000
100
10
1
8:31:39 AM
9:56:39 AM
10:51:39 AM
11:48:39 AM
12:43:39 PM
1:38:39 PM
2:33:39 PM
3:28:39 PM
4:44:06 PM
5:52:06 PM
6:47:06 PM
7:42:06 PM
8:37:06 PM
9:32:06 PM
10:27:06 PM
11:22:06 PM
12:17:06 AM
1:12:06 AM
2:07:06 AM
3:02:07 AM
3:57:07 AM
4:52:07 AM
5:47:07 AM
6:42:07 AM
7:37:07 AM
9:26:07 AM
10:35:07 AM
11:51:07 AM
1:12:07 PM
2:07:07 PM
3:09:07 PM
5:34:00 PM
6:31:00 PM
7:26:00 PM
8:21:00 PM
9:16:00 PM
10:11:00 PM
11:06:00 PM
12:01:00 AM
12:56:00 AM
1:51:00 AM
2:46:00 AM
3:41:00 AM
4:36:00 AM
5:31:00 AM
6:26:00 AM
7:21:00 AM
8:24:00 AM
9:37:00 AM
10:46:00 AM
12:18:00 PM
1:27:00 PM
2:30:00 PM
3:25:00 PM
Outdoor Air Intake
Recovery Suite
Supply Air
Workshift

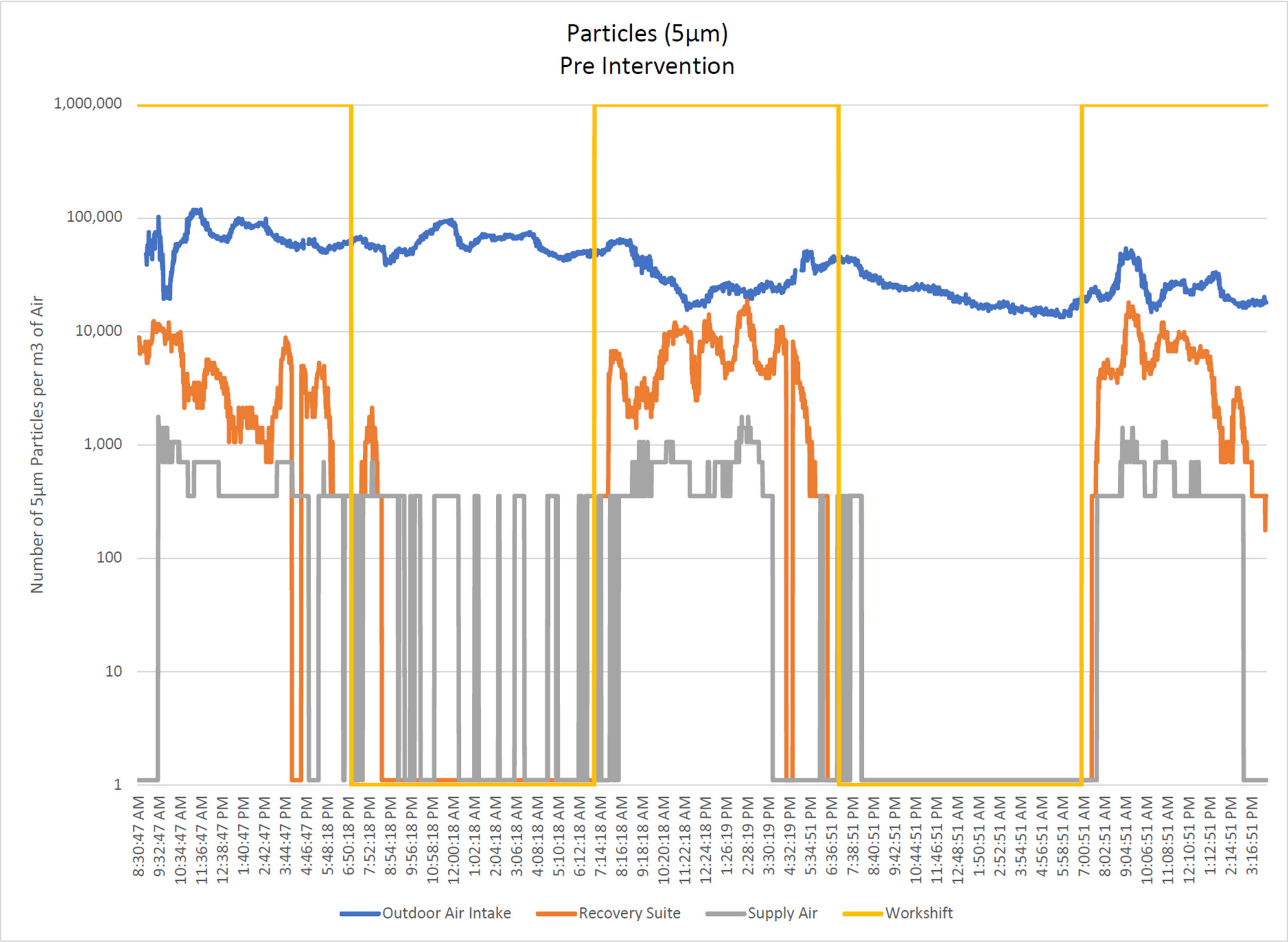

Particles (5µm)
Pre Intervention
Number of 5µm Particles per m3 of Air
1,000,000
100,000
10,000
1,000
100
10
1
Outdoor Air Intake
Recovery Suite
Supply Air
Workshift

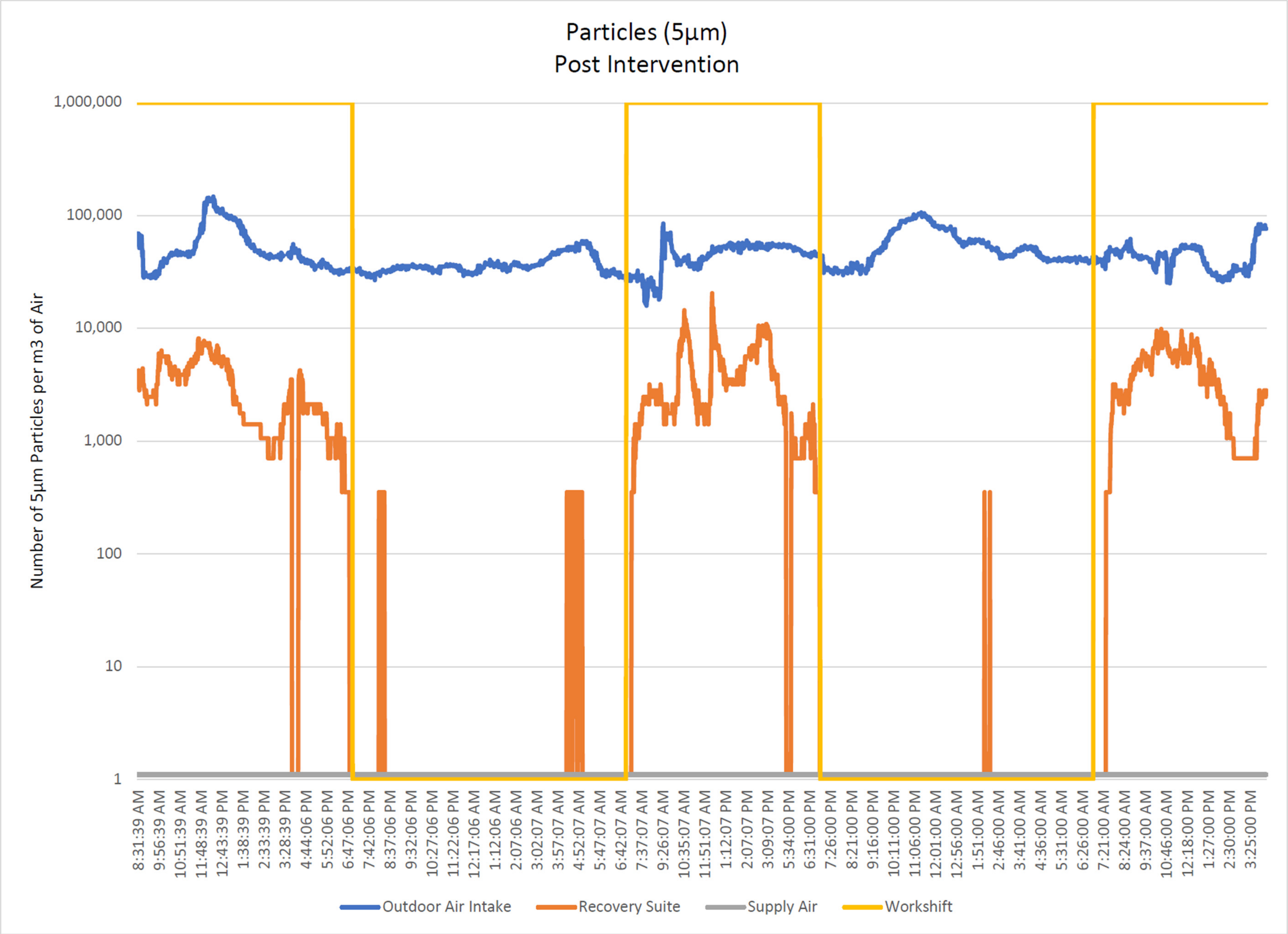

Particles (5µm)
Post Intervention
Number of 5µm Particles per m3 of Air
1,000,000
100,000
10,000
1,000
100
10
1
8:31:39 AM
9:56:39 AM
10:51:39 AM
11:48:39 AM
12:43:39 PM
1:38:39 PM
2:33:39 PM
3:28:39 PM
4:44:06 PM
5:52:06 PM
6:47:06 PM
7:42:06 PM
8:37:06 PM
9:32:06 PM
10:27:06 PM
11:22:06 PM
12:17:06 AM
1:12:06 AM
2:07:06 AM
3:02:07 AM
3:57:07 AM
4:52:07 AM
5:47:07 AM
6:42:07 AM
7:37:07 AM
9:26:07 AM
10:35:07 AM
11:51:07 AM
1:12:07 PM
2:07:07 PM
3:09:07 PM
5:34:00 PM
6:31:00 PM
7:26:00 PM
8:21:00 PM
9:16:00 PM
10:11:00 PM
11:06:00 PM
12:01:00 AM
12:56:00 AM
1:51:00 AM
2:46:00 AM
3:41:00 AM
4:36:00 AM
5:31:00 AM
6:26:00 AM
7:21:00 AM
8:24:00 AM
9:37:00 AM
10:46:00 AM
12:18:00 PM
1:27:00 PM
2:30:00 PM
3:25:00 PM
Outdoor Air Intake
Recovery Suite
Supply Air
Workshift

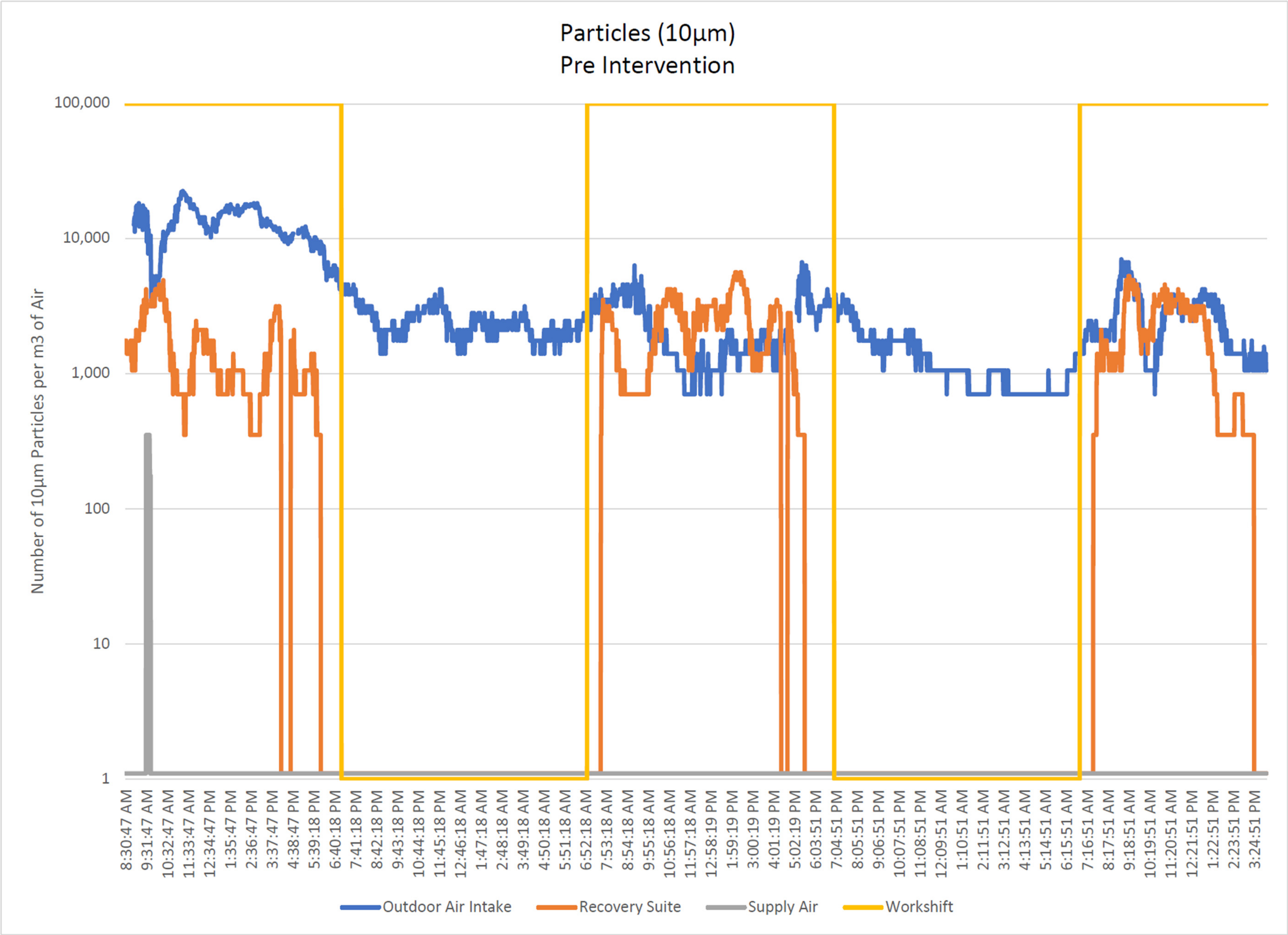

Particles (10µm)
Pre Intervention
Number of 10µm Particles per m3 of Air
100,000
10,000
1,000
100
10
1
8:30:47 AM
9:31:47 AM
10:32:47 AM
11:33:47 AM
12:34:47 PM
1:35:47 PM
2:36:47 PM
3:37:47 PM
4:38:47 PM
5:39:18 PM
6:40:18 PM
7:41:18 PM
8:42:18 PM
9:43:18 PM
10:44:18 PM
11:45:18 PM
12:46:18 AM
1:47:18 AM
2:48:18 AM
3:49:18 AM
4:50:18 AM
5:51:18 AM
6:52:18 AM
7:53:18 AM
8:54:18 AM
9:55:18 AM
10:56:18 AM
11:57:18 AM
12:58:19 PM
1:59:19 PM
3:00:19 PM
4:01:19 PM
5:02:19 PM
6:03:51 PM
7:04:51 PM
8:05:51 PM
9:06:51 PM
10:07:51 PM
11:08:51 PM
12:09:51 AM
1:10:51 AM
2:11:51 AM
3:12:51 AM
4:13:51 AM
5:14:51 AM
6:15:51 AM
7:16:51 AM
8:17:51 AM
9:18:51 AM
10:19:51 AM
11:20:51 AM
12:21:51 PM
1:22:51 PM
2:23:51 PM
3:24:51 PM
Outdoor Air Intake
Recovery Suite
Supply Air
Workshift

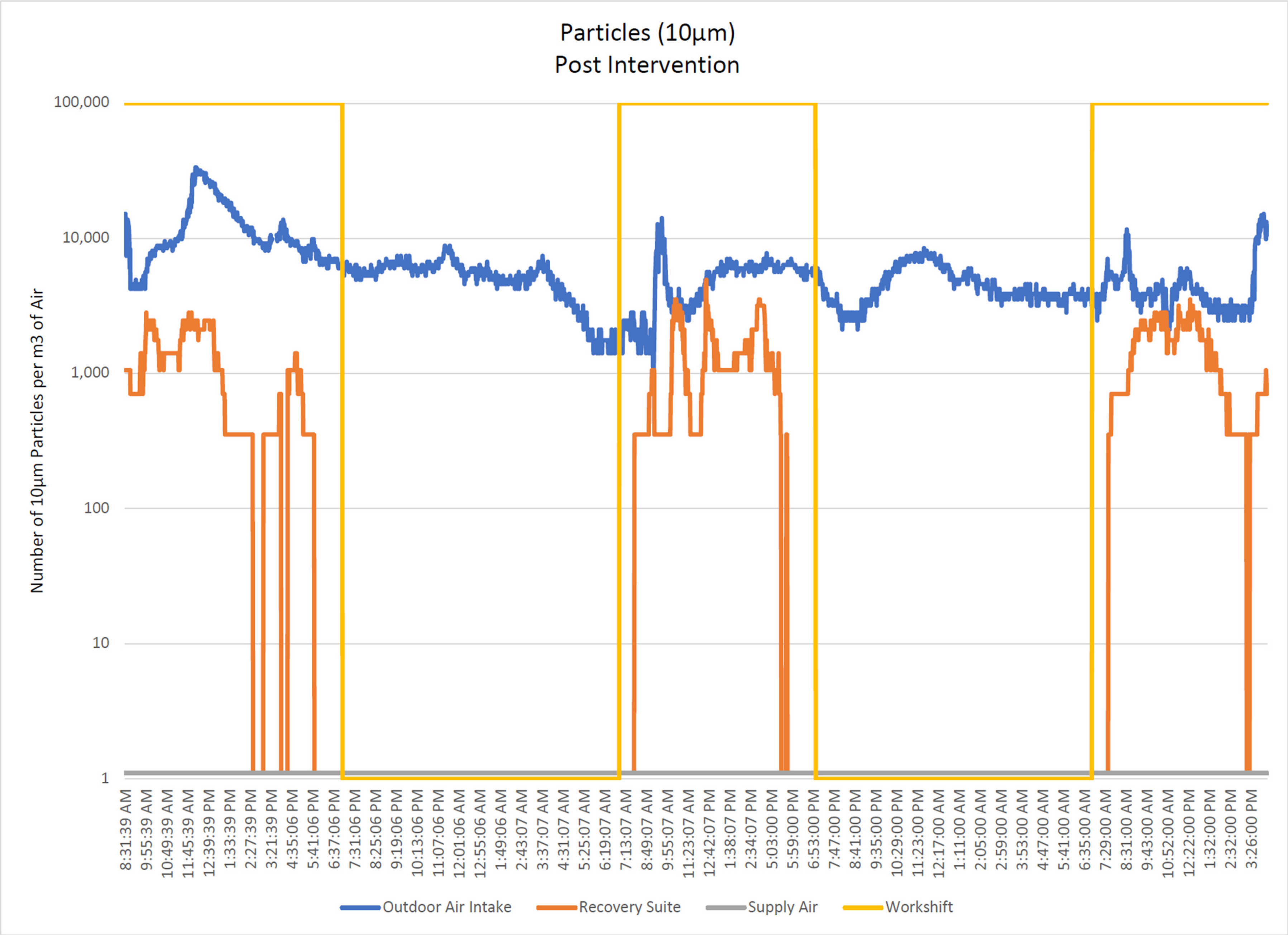
Particles (10µm)
Post Intervention
Number of 10µm Particles per m3 of Air
100,000
10,000
1,000
100
10
1
8:31:39 AM
9:55:39 AM
10:49:39 AM
11:45:39 AM
12:39:39 PM
1:33:39 PM
2:27:39 PM
3:21:39 PM
4:35:06 PM
5:41:06 PM
6:37:06 PM
7:31:06 PM
8:25:06 PM
9:19:06 PM
10:13:06 PM
11:07:06 PM
12:01:06 AM
12:55:06 AM
1:49:06 AM
2:43:07 AM
3:37:07 AM
4:31:07 AM
5:25:07 AM
6:19:07 AM
7:13:07 AM
8:49:07 AM
9:55:07 AM
11:23:07 AM
12:42:07 PM
1:38:07 PM
2:34:07 PM
5:03:00 PM
5:59:00 PM
6:53:00 PM
7:47:00 PM
8:41:00 PM
9:35:00 PM
10:29:00 PM
11:23:00 PM
12:17:00 AM
1:11:00 AM
2:05:00 AM
2:59:00 AM
3:53:00 AM
4:47:00 AM
5:41:00 AM
6:35:00 AM
7:29:00 AM
8:31:00 AM
9:43:00 AM
10:52:00 AM
12:22:00 PM
1:32:00 PM
2:32:00 PM
3:26:00 PM
Outdoor Air Intake
Recovery Suite
Supply Air
Workshift

Table S1: Mean and standard deviation (SD) of particles numbers by channel size (0.3, 0.5, 1, 3, 5 and 10 μm /m³ air) during pre and post intervention for outdoor, supply and recovery room air, summarised by the total time, by work hours and by night. Particle number concentrations are divided by 10³.

The mean particle number concentrations and their standard deviation per channel size are depicted for the total time during pre-intervention and post-intervention, during work hours, and during night-time. Pre-intervention particle number concentrations are substantially reduced in the supply air and recovery room air compared to the outdoor air. Reductions in particles compared to outside air indicate that the pre-intervention filter (MERV-13) is successful for particle channels 3,5 and 10, but less for size 0.3, 0.5 and 1. Post intervention, the reduction in both supply and recovery room air particles compared to outdoor air is significantly greater for particle channels 0.3, 0.5 and 1 than during pre-intervention indicating that the intervention device is working well for sub-micron particles.

**Pre-Intervention**													
		0.3 um (particles/m³)		0.5 um (particles/m³)		1 um (particles/m³)		3 um (particles/m³)		5 um (particles/m³)		10 um (particles/m³)	
	Sample N	Mean	SD	Mean	SD	Mean	SD	Mean	SD	Mean	SD	Mean	SD
	**Outdoor Air**												
Total	2757	11,802	11,109	2,739	1,493	1,784	1,233	106	61	45	24	4	4
Work	1328	7,194	5,794	1,644	1,003	1,047	656	82	34	49	25	7	5
Night	1429	15,907	12,956	3,715	1,079	2,441	1,241	127	70	41	22	1	1
	**Supply Air**												
Total	2757	1,554	1,281	331	174	57	30	1	1	0.2	0.3	0.001	0.002
Work	1328	1,162	766	234	137	48	24	1	1	0.4	0.3	0.003	0.003
Night	1429	1,867	1,504	408	144	64	31	0.6	0.3	0.1	0.2	0	0

	**Recovery Room Air**												
Total	2757	1,060	752	231	103	49	28	2	2	2	3	0.6	1
Work	1328	886	489	194	92	58	33	3	2	3	3	1	1
Night	1429	1,216	899	264	98	41	21	0.2	0.3	0.05	0.2	0	0
						**Post-Intervention**							
	**Outdoor Air**												
Total	2961	3,562	1,560	2,463	1,461	1,520	951	90	42	47	19	6	4
Work	1526	3,251	1,120	2,215	973	1,332	583	85	27	48	20	7	5
Night	1435	3,891	1,861	2,725	1,805	1,718	1,194	96	52	45	17	4	2
	**Supply Air**												
Total	2961	2	3	0.8	0.9	0.02	0.01	0	0	0	0	0	0
Work	1526	3	5	1	1	0.05	0.1	0	0	0	0	0	0
Night	1435	1	0.7	0.4	0.5	0	0	0	0	0	0	0	0
	**Recovery Room Air**												
Total	2961	74	112	38	34	21	23	2	4	2	3	0.5	0.8
Work	1526	108	148	54	40	34	25	4	4	3	3	1	1
Night	1435	38	14	21	10	7	3	0.7	0.1	0.01	0.06	0	0
